# Structural definition of a novel CD4-induced epitope that is targeted by a single-headed immunoglobulin to effect broad and potent HIV neutralization

**DOI:** 10.1186/1742-4690-9-S2-P346

**Published:** 2012-09-13

**Authors:** P Acharya, TS Luongo, J Matz, SD Schmidt, G Chuang, I Georgiev, P Kessler, Y Yang, P Chames, L Martin, JR Mascola, PD Kwong

**Affiliations:** 1 National Institutes of Health, Bethesda, MD, USA; 2 Inserm, Marseille, France; 3 CEA/iBiTecs, Gif sur Yvette, France; 4 INSERM, Marseille, France

## Background

HIV-1 enters cells by sequentially binding the CD4 receptor and a coreceptor, either CCR5 or CXCR4. Functional constraints result in a high degree of conservation of the receptor-binding sites making them potential targets for intervention. The coreceptor-binding site on HIV-1 envelope gp120 glycoprotein is protected from the humoral immune system by conformational masking and steric occlusion. The site becomes available after a conformational change in gp120 following CD4 engagement, but at that point in the entry process, the proximity of the viral and cellular membranes makes the site inaccessible to bulky antibody molecules. Thus, in spite of being highly conserved, this region has not been considered a viable vaccine target.

## Methods

Single domain antibody vHH120.4 was isolated from llama immunized with gp120 covalently linked to a CD4-mimetic peptide. Full-length versions of this antibody (IgG2B and IgG3) were created and tested for neutralization. The structure of vHH120.4 bound to gp120 from the HIV-1 YU2 strain was determined at 2.1Å resolution.

## Results

Both IgG2B and IgG3 versions of the vHH120.4 potently neutralized over 95% of a panel of circulating Tier 2 HIV-1 isolates. Structural analyses of vHH120.4 bound to gp120 revealed a novel CD4i epitope that involves antibody interactions with region on gp120 encompassing the bridging sheet and the base of the V3 loop, the β19 strand, the CD4-binding loop, and the glycan at Asn 386. This epitope overlaps the classically defined CD4-induced epitopes recognized by antibodies 17b and 48d, but is shifted towards the site of CD4 attachment.

## Conclusion

The discovery of a neutralizing CD4-induced epitope indicates that not all CD4-induced sites are masked from neutralization. Whether human antibodies can also utilize the newly defined vHH120.4 epitope for effective neutralization remains to be determined; HIV-1 envelope probes designed to specifically select antibodies targeting this epitope are now being developed.

